# Endometriosis, a common but enigmatic disease with many faces: current concept of pathophysiology, and diagnostic strategy

**DOI:** 10.1007/s11604-024-01569-5

**Published:** 2024-04-25

**Authors:** Mayumi Takeuchi, Kenji Matsuzaki, Masafumi Harada

**Affiliations:** 1 Department of Radiology, Tokushima University, 3-18-15, Kuramoto-Cho, Tokushima, 7708503 Japan; 2 Department of Radiological Technology, Tokushima Bunri University, Sanuki City, ShidoKagawa, 1314-17692193 Japan

**Keywords:** Endometriosis, Deep endometriosis, Endometrioma, Malignant transformation, Magnetic resonance imaging (MRI)

## Abstract

Endometriosis is a benign, common, but controversial disease due to its enigmatic etiopathogenesis and biological behavior. Recent studies suggest multiple genetic, and environmental factors may affect its onset and development. Genomic analysis revealed the presence of cancer-associated gene mutations, which may reflect the neoplastic aspect of endometriosis. The management has changed dramatically with the development of fertility-preserving, minimally invasive therapies. Diagnostic strategies based on these recent basic and clinical findings are reviewed. With a focus on the presentation of clinical cases, we discuss the imaging manifestations of endometriomas, deep endometriosis, less common site and rare site endometriosis, various complications, endometriosis-associated tumor-like lesions, and malignant transformation, with pathophysiologic conditions.

## Introduction

Endometriosis significantly impairs women's quality of life with chronic pain and reduced fertility and has become an important issue from a public health perspective in light of recent lifestyle changes. Endometriosis is a benign, common, but controversial disease due to its enigmatic etiopathogenesis and biological behavior. Recent studies suggest multiple genetic, and environmental factors such as endocrine, inflammatory, immunological, and angiogenetic may affect its onset and development [1–3]. Genomic analysis revealed the presence of cancer-associated gene mutations, which may reflect the neoplastic aspect of endometriosis [1, 3].

Endometriosis affects approximately 10% of women during their reproductive years, meaning that it affects approximately 190 million women worldwide [1, 2]. Endometriosis is a chronic, inflammatory disease characterized by the presence of endometrial-like tissue outside the uterus, and is associated with debilitating painful symptoms in many patients, also at greater risk of infertility [3–6], emergence of fatigue, multisite pain, and other comorbidities. High risk of subsequent development of autoimmune disease, cancer, and cardiovascular disease is also reported [1, 7].

In this article, we reviewed the imaging features of endometriosis and various related conditions.

## Causes of endometriosis

In retrograde menstruation, menstrual blood containing endometrial cells with mutation (*ARID1A/PIK3CA*) flows back through the fallopian tubes and into the pelvic cavity [1, 8–10]. These endometrial cells stick to the pelvic walls and surfaces of the pelvic organs, where they grow and continue to thicken and bleed for each menstrual cycle. Endometrioma (endometriotic cyst) may be formed in the ovaries [4, 8, 9, 11].

Other causes implicated in endometriosis include coelomic metaplasia, in which hormonal or immune factors facilitate the conversion of peritoneal cells into endometrial tissue; embryonic cell transformation, in which estrogen could induce the conversion of embryonic cells into endometrial tissue during puberty; surgical scar implantation, in which endometrial cells may adhere to surgical incisions post-surgery, such as after a cesarean section; endometrial cell transport, in which blood vessels or the lymphatic system might ferry endometrial cells to distant sites; and immune system disorders, which may prevent the body from identifying and eliminating endometrial tissue growing outside the uterus [3, 4, 11].

## Classification of endometriosis

The revised American Society for Reproductive Medicine (rASRM) classification based on laparoscopic findings is accepted globally and has been widely used [12]. The rASRM is staged according to a point system based on the presence, size (< 1 cm, 1–3 cm, or > 3 cm), and depth (superficial or deep) of ovarian and peritoneal endometriosis, the presence and extent of ovarian and tubal adhesions (filmy or dense), and the degree of posterior cul-de-sac obliteration (partial or complete). The rASRM is easy to explain the degree of endometriosis in simple terms to patients. Disadvantages of rASRM are (1) Difference between histology and laparoscopic diagnosis, (2) The reproducibility is poor, (3) Infertility and severities of pain are not correlated, and (4) The presence of deep endometriosis is not considered [13, 14].

ENZIAN classification is based on laparoscopic findings but could be determined by imaging modalities (US and MRI) [15]. The advantages of ENZIAN classification are (1) A comprehensive minimally invasive and surgical description system for endometriosis, (2) It provides detailed descriptions of the retroperitoneal structures, (3) It can be determined by imaging modalities such as US and MRI, and (4) Localization and extent of endometriosis are associated and correlated with the presence and severity of different symptoms. MRI–ENZIAN correlation has a high accuracy of 95 to 97% for deep endometriosis. However, it has some disadvantages: (1) International acceptance is poor, (2) Patients may not readily understand, (3) Inaccurate if incomplete surgical dissection of deep endometriosis or image study alone is performed, 4) There are not sufficient studies regarding the feasibility of the classification determined by imaging [14, 16–18].

## Endometrioma (endometriotic cyst)

**Fig. 1:**
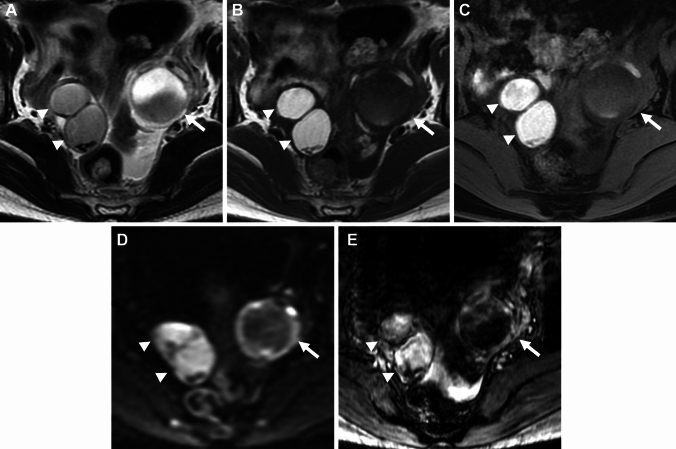
Endometrioma (typical) and hemorrhagic corpus luteum cyst. A 40-year-old with a history of pelvic pain and suspicion of bilateral ovarian cysts on ultrasonography examination. **A** T2-weighted image reveals bilateral ovarian cysts. The right cysts (arrowheads) show a faint, layered signal loss, whereas the left cyst (arrow) shows a partial signal loss. T2 dark spots are demonstrated in the right posterior cyst. **B** On T1-weighted image, the right cysts show high signal intensity (≥ fat) and the left cyst exhibits low signal intensity. No signal decrease is observed in the right ovarian cysts on **C** fat-saturated T1-weighted image. Multiple T1-high signal intensity cysts (multiplicity), the shading sign (T2-shortening in adnexal cyst exhibiting T1-high signal intensity), and the presence of T2 dark spot suggest right ovarian endometriomas. The left cyst is suggestive of the non-endometriotic hemorrhagic cyst (corpus luteum cyst). **D** Diffusion-weighted image (DWI) (*b* = 800 s/mm^2^) shows totally high signal intensity in the right cysts, and ring-like high signal intensity in the left cyst. **E** Susceptibility-weighted image (SWAN: susceptibility-weighted angiography) reveals dotty to curved linear signal voids in the walls of the right cysts. Signal voids are prominent within the left cysts but not in the cyst wall

Endometriosis is usually seen in the pelvic cavity, especially in the peritoneum, uterine ligaments (the uterosacral, broad, or round), and ovaries. Less commonly, the bowel, ureter, bladder, lymph nodes, and cesarean section scar may be affected. Ovarian endometriomas (endometriotic cysts) are characterized by altered luminal blood as “chocolate cysts” caused by repeated cyclic hemorrhage [4, 5]. Multiple high signal intensity cysts on T1-weighted images (multiplicity), and the shading sign (T2-shortening in adnexal cyst exhibiting T1-high signal intensity) are suggestive findings of endometrioma (Fig. 1) [19–21]. “Shading” may appear as a complete loss of signal or dependent layering with a low signal intensity fluid level on T2-weighted images. The cause of T2-low signal intensity is complex; hyperviscosity and high concentration of protein and hemosiderin from recurrent cyclical bleeding may contribute to T2-shortening [22]. The overall diagnostic value obtained using shading sign (T1-high: ≥ fat) and/or multiplicity is a sensitivity of 90%, specificity of 98%, and accuracy of 96% [20]. A follow-up study reported better sensitivity (93%) but low specificity (45%) [23], however, this discrepancy may be due to differences in signal threshold in T1-weighted image (T1-high: ≥ skeletal muscle). Lesions that do not show as strong a signal as fat on T1-weighted images may include non-endometrial hemorrhagic cysts, suggesting that specificity may have decreased. Doubling of magnetic susceptibility at 3 T may influence the diagnostic ability. Because susceptibility-induced signal intensity loss may increase from 1.5 T to 3 T, the shading sign is well visualized at 3 T [24]. T2 dark spot sign (T2-low signal intensity clots within the cyst which are often linear, punctate, or oval in shape) is specific for endometrioma with a sensitivity of 36% and specificity of 93% [23].

**Fig. 2:**
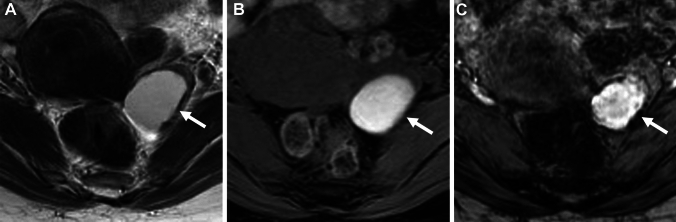
Endometrioma. A 45-year-old with a history of dysmenorrhea and suspicion of a left ovarian cyst on ultrasonography examination. **A** T2-weighted image reveals a high signal intensity left ovarian cyst (arrow). **B** On fat-saturated T1-weighted image, the left ovarian cyst (arrow) shows high signal intensity. **C** SWAN reveals dotty to curved linear signal voids in the cyst wall (arrow), which suggest endometrioma

Deposition of hemosiderin-laden macrophages within the cyst wall due to repeated cyclic hemorrhage is a pathologic feature of endometrioma, and dotty or curved linear signal voids due to hemosiderin deposition along the cyst wall on susceptibility-weighted images (SWI) are suggestive of endometrioma (Figs. 1, 2) [25, 26]. One study reported an overall diagnostic value of accuracy of 97.6% with the combination of the shading sign and SWI [25]. Because signal voids within the cyst may be seen also in non-endometriotic lesions such as hemorrhagic corpus luteum cysts, the presence of signal voids in the cyst wall should be carefully interpreted [26]. Signal voids due to hemosiderin-deposition along the walls of endometriomas on SWI are more prominent at 3 T than at 1.5 T, however, susceptibility artifacts caused by intestinal gas, metallic, or other materials also are more prominent at 3 T [25].

## Deep endometriosis/extragenital endometriosis

### Deep endometriosis

**Fig. 3:**
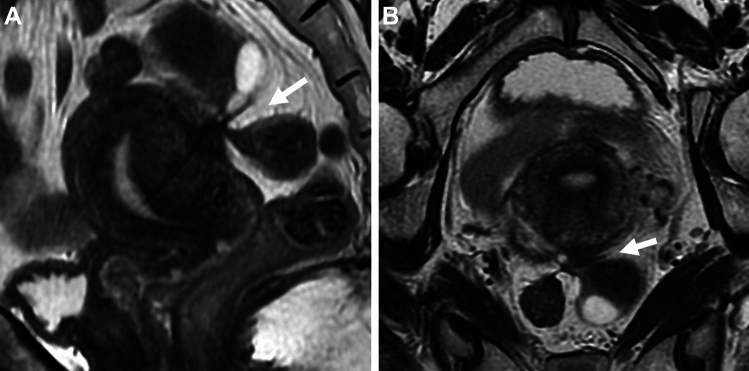
Deep endometriosis. A 48-year-old with a history of pelvic pain and suspicion of deep posterior endometriosis. **A** Sagittal and **B** oblique coronal T2-weighted images show the elevation of the posterior vaginal fornix, low signal intensity fibrotic plaque with stellate margins (arrow) on the serosal surface of the retroflexed uterus. Tethered appearance of the rectum to the uterus with low signal intensity faint fibrous strands is seen

Deep (infiltrating) endometriosis may cause severe pelvic pain and infertility and is classically defined by the invasion of endometrial tissue at least 5 mm beneath the peritoneal surface [27]. Clinically, “adenomyosis externa” at the rectosigmoid, Douglas' pouch, uterosacral ligament, rectovaginal septum, vesico-uterine pouch, etc. is considered deep endometriosis [28]. Deep endometriosis of the peritoneum, ligaments, or organs may cause solid masses exhibiting T2-low signal intensity and intense contrast enhancement reflecting fibromuscular hyperplasia around sparse ectopic endometrial glands (Fig. 3) [29–35]. The cyclic hemorrhage of the ectopic endometrial tissue may cause a variable inflammatory response and fibrous reaction. The endometrial tissue infiltrates the adjacent fibromuscular tissue and induces smooth muscle proliferation and fibrous reaction, resulting in the formation of T2-low signal intensity solid masses as “adenomyosis extern” with irregular, indistinct, or stellate margins, or T2-low signal intensity soft tissue thickening [29–35]. T1-high signal intensity hemorrhagic foci and SWI-signal voids due to hemosiderin deposits may be observed. In one study, 89% of deep endometriosis was revealed on SWI whereas 61% on T1-weighted images [36]. In evaluating posterior cul-de-sac obliteration due to deep endometriosis, retroflexed uterus, elevated posterior vaginal fornix, intestinal tethering and/or a tethered appearance of the rectum in the direction of the uterus, faint strands between the uterus and intestine, and fibrotic plaque and/or nodule covering the serosal surface of the uterus are specific MR findings [37]. In addition, displacement of intraperitoneal fluid is another suggestive finding of posterior cul-de-sac obliteration due to endometriosis [38]. T2-low signal intensity faint strands reflecting fibrotic adhesions should be interpreted with caution in mild cases.

### The kissing ovaries sign/cloverleaf sign

**Fig. 4:**
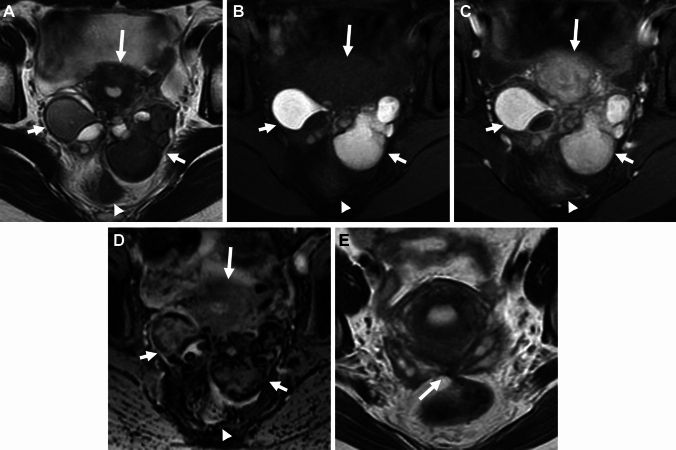
Deep endometriosis (kissing ovaries /cloverleaf sign). A 27-year-old with a history of irregular menstruation and suspicion of bilateral ovarian cysts on ultrasonography examination. **A** On T2-weighted image and **B** fat-saturated T1-weighted image, bilateral endometriomas (short arrows) are observed posterior to the uterus (long arrow), appearing as “kissing ovaries” with extensive fibrous adhesions exhibit low signal intensity on T2-weighted image and intense contrast enhancement on **C** post-contrast fat-saturated T1-weighted image. A tethered appearance of the rectum (arrowhead) with T2-low signal intensity faint fibrous strands is observed. Small hemorrhagic foci within deep endometriosis are scattered as T1-high signal intensity spots and spotty signal voids on **D** SWI. Curved linear signal voids along the cyst wall are observed on SWI. A 36-year-old with deep endometriosis shows the cloverleaf sign as both ovaries, uterus, and rectum coming together in the center of the figure formed by low signal intensity constrictive adhesions (arrow) on **E** T2-weighted image

Both ovaries may be located close to or are touching each other in the pouch of Douglas referred to as the kissing ovaries sign, usually due to pelvic adhesions (Fig. 4A–D). This finding is strongly related to disease severity and frequency of infertility, and has a significant correlation with deep endometriosis with a sensitivity of 67%, specificity of 68%, and positive predictive value (PPV) of 55% [39]. Occasionally “cloverleaf sign” may be observed: the “leaves” are formed by at least three different organs such as both ovaries, uterus, or rectum, coming together in the center of the figure formed by constrictive adhesions (Fig. 4E) [40].

### Torus uterinus/uterosacral ligament/round ligament

**Fig. 5:**
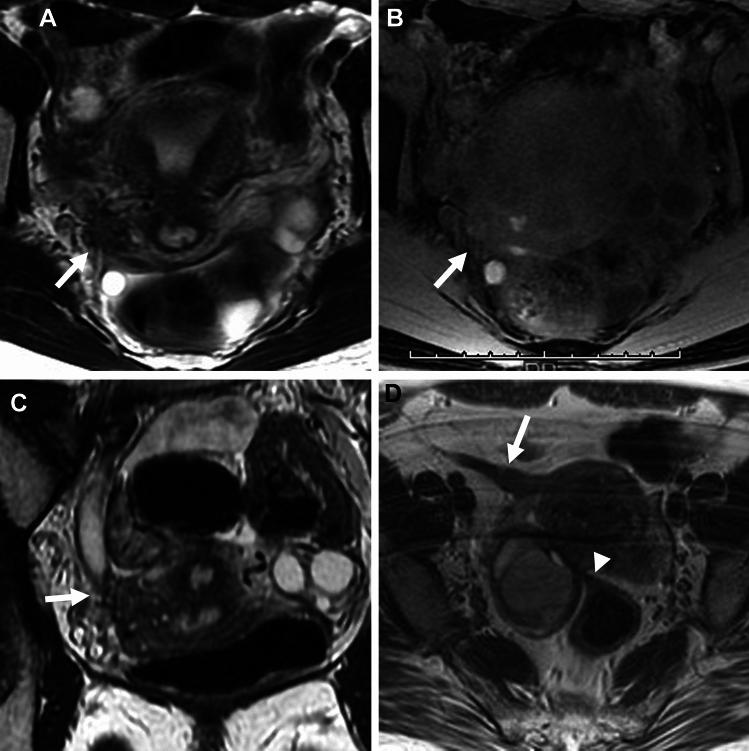
Deep endometriosis (rectosigmoid/round ligaments). A 29-year-old with a history of dysmenorrhea. **A** T2-weighted image and **B **fat-saturated T1-weighted image reveal a fibrotic plaque (arrow) on the right posterior serosal surface of the uterus to the right uterosacral ligament. Mottled T1-high signal intensity hemorrhagic foci are observed. **C** The right ureter is involved (arrow) resulting in right hydronephrosis on coronal T2-weighted image. A 46-year-old with right endometrioma. **D** On T2-weighted image, the right thickened round ligament (arrow) and a tethered appearance of the rectum to the uterus with low signal intensity faint fibrous strands (arrowhead) are revealed

Torus uterinus is anatomically defined by the presence of a small transverse thickening that binds the original insertion of uterosacral ligaments on the posterior wall of the uterus. Torus uterinus and uterosacral ligaments are the most frequent sites of deep endometriosis (86%) [34]. Torus uterinus involvement may appear as a T2-low signal intensity mass or thickening in the upper middle portion of the posterior cervix, and involvement of the uterosacral ligament with endometriosis may bore a nodule with regular or stellate margins or show fibrotic thickening (Fig. 5A–C). Uterosacral ligament involvement may often be asymmetric, unilateral in 40%, and bilateral in 60% [34]. The round ligament involvement is less common (3–5%) with left-side predominance. The round ligament is identified as thin structures running from the uterine horns to the pelvic sidewall. The involved ligament may appear thickened, shortened, and irregular, with a nodular appearance (Fig. 5D) [41, 42]. The distal part of the round ligament is situated extra-pelvic site and may appear as a painful, palpable inguinal mass, with menstrual variation in the size or severity of symptoms [43].

### Focal adenomyosis located in the outer myometrium (FAOM)

**Fig. 6:**
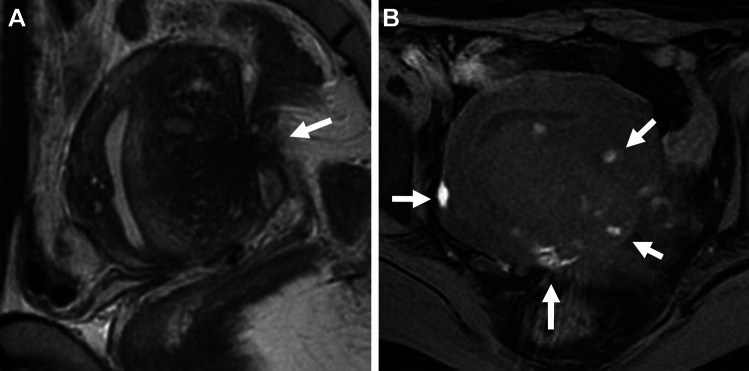
Focal adenomyosis located in the outer myometrium (FAOM). A 39-year-old with a history of dysmenorrhea and hypermenorrhea. **A** On sagittal T2-weighted image ill-defined low signal intensity area is revealed in the thickened posterior myometrium separated from the junctional zone. Low signal intensity fibrous plaque with adhesive change (arrow) is seen posterior to the myometrial lesion. **B** On fat-saturated T1-weighted image high signal intensity hemorrhagic spots (arrows) are scattered

Focal adenomyosis located in the outer myometrium (FAOM) is observed more frequently in women with endometriosis and was significantly associated with deep endometriosis [44–46]. FAOM is separated from the junctional zone which was kept intact and with preserved healthy muscular structures between the adenomyosis and the junctional zone on T2-weighted images (Fig. 6) [44]. FAOM may be caused by endometriotic invasion from the outside, whereas usual adenomyosis is a product of direct endometrial invasion [44–46].

### Urinary tract endometriosis

**Fig. 7:**
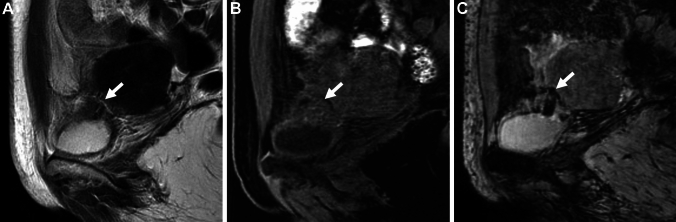
Bladder endometriosis. A 42-year-old with frequent urination during menstruation. **A** Sagittal T2-weighted image shows low signal intensity mass (arrow) at the posterior wall of the bladder. Small hemorrhagic foci are scattered in the mass (arrow) as high signal intensity spots on **B** sagittal fat-saturated T1-weighted image and dotty signal voids on **C** sagittal SWAN

Urinary tract involvement is rare and occurs in only about 1% of endometriosis. Bladder endometriosis is the most common, and rarely ureteral involvement may occur usually with hydronephroureter. The posterior wall and the dome are commonly affected areas of bladder endometriosis [41, 47, 48]. Less than 30% of patients suffer from cyclical hematouria, because endometrial deposits are usually submucosal and mucosal infiltration is relatively rare. Bladder endometriosis may appear as a T2-low signal intensity solid mass reflecting fibromuscular hyperplasia. T1-high signal intensity small hemorrhagic foci may be observed within the mass (Fig. 7) [47]. Signal voids due to hemosiderin deposits reflecting repeated cyclic hemorrhage on SWI are characteristic, and more sensitive than T1-weighted images [49].

Extra-peritoneal involvement of endometriosis may cause adhesive stricture of the ureter resulting in hydronephroureter. Preoperative diagnosis may be often difficult because it may occur with no obvious mass formation, and thin slice T2-weighted images can reveal low signal intensity fibrous adhesion [41, 48]. DWI could differentiate endometriosis-related hydronephroureter from ureteral cancer. Cancer appears as a DWI-high signal intensity lesion, whereas endometriosis does not cause a signal increase on DWI.

### Bowel endometriosis

**Fig. 8:**
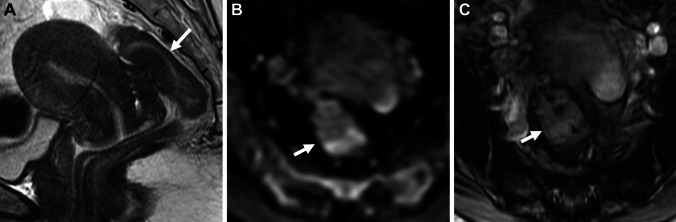
Bowel endometriosis. A 47-year-old with hematochezia during menstruation. **A** On sagittal T2-weighted image, the anterior wall of the rectosigmoid colon is thickened exhibiting low signal intensity covered by high signal intensity mucosa and submucosa as "mushroom cap" shaped appearance (arrow). Low signal intensity fibrous strands between the uterus and rectosigmoid colon are observed. **B** DWI (*b* = 800 s/mm^2^), **C** SWAN. The mass-like thickened wall (arrow) shows no diffusion restriction on DWI. Small hemorrhagic foci are scattered as dotty signal voids on SWAN

Rectosigmoid (65.7%) is the most common site of deep endometriosis involving bowels, followed by ileocecal junction (20%) and rectum (15%). Endometrial implants adhere to the bowel serosa and invade the muscle layers with marked smooth muscle proliferation, consequently, irregular bowel wall thickening with stricture formation resembling carcinoma. No mucosal involvement is a helpful finding to differentiate from cancer on barium enema or colonoscopy [41, 50–52]. The involved bowel wall may appear as a T2-low signal intensity mass reflecting fibromuscular hyperplasia with small hemorrhagic foci revealed as T1-high signal intensity spots and/or SWI-signal voids (Fig. 8) [49, 50]. "Mushroom cap" shaped appearance is characteristic of rectosigmoid endometriosis: heterogeneous T2-low signal intensity hypertrophic muscularis propria, covered with T2-high signal intensity mucosa and submucosa (Fig. 8A) [51].

### Retroperitoneal endometriosis (lymph node involvement)

**Fig. 9:**
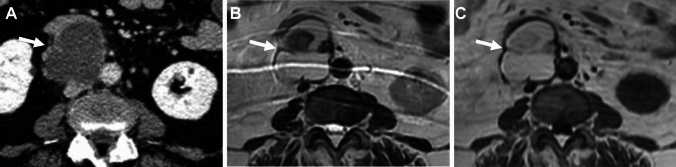
Retroperitoneal endometriosis (lymph node involvement). A 72-year-old with suspicion of abdominal mass on ultrasonography examination. **A** Contrast-enhanced CT reveals a cystic mass (arrow) located in the retroperitoneal space. The mass shows high signal intensity on both **B** T2-weighted image and **C** T1-weighted image suggesting its hemorrhagic contents with clots

Pelvic endometriosis may often involve adjacent retroperitoneal space, however, extra-pelvic retroperitoneal endometriosis is rare. The pathogenesis is thought to be secondary to retrograde menstruation, vascular or lymphatic spread, or coelomic metaplasia, and lymphatic spread is the most possible cause. Occasionally retroperitoneal lymph nodes contain endometrial tissue (Fig. 9) [41, 53, 54].

### Abdominal wall/subcutaneous endometriosis

**Fig. 10:**
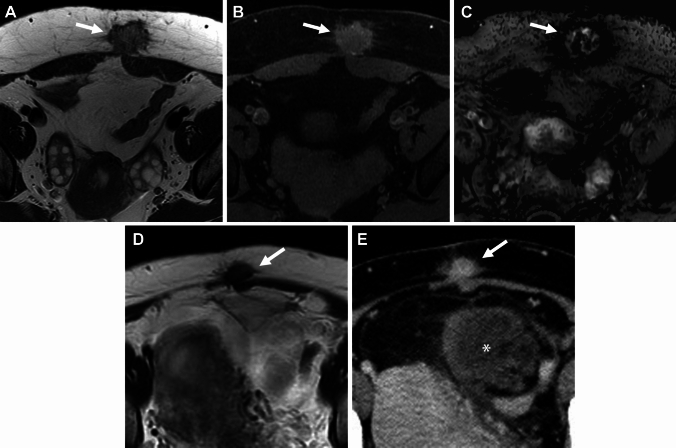
Abdominal wall endometriosis. A 34-year-old with lower abdominal discomfort during menstruation. **A** On T2-weighted image, a subcutaneous mass with irregular margins (arrow) exhibits heterogeneous signal intensity at the previous cesarean surgical scar. Small high signal intensity hemorrhagic foci are revealed on **B** fat-saturated T1-weighted image. Prominent signal voids are observed in and around the mass (arrow) on **C** SWI. A 37-year-old with lower abdominal discomfort during menstruation. **D** On T2-weighted image a subcutaneous low signal intensity mass (arrow) at the previous cesarean surgical scar is revealed. **E** On CT, linear infiltration irradiating peripherally from a central soft tissue mass (arrow) as the gorgon sign is observed. *: Concomitant mesenteric liposarcoma

Abdominal wall endometriosis usually develops in association with previous surgical scars such as cesarean section, but spontaneous abdominal involvement may also occur. Cyclic abdominal discomfort or pain with a palpable mass may suggest abdominal wall endometriosis. Inhomogeneous signal intensity mass on T2-weighted images due to the admixture of fibrosis, hemorrhage, and endometrial tissue, T1-high signal intensity spotty hemorrhage, and SWI-signal voids due to hemosiderin deposits are helpful for the diagnosis (Fig. 10) [41, 49, 55–58]. Linear infiltration irradiating peripherally from a central soft tissue nodule as the gorgon sign is suggestive CT finding (Fig. 10E) [55]. Subcutaneous endometriosis may occasionally appear as an endometrioma-like hemorrhagic cystic mass.

### Thoracic endometriosis

**Fig. 11:**
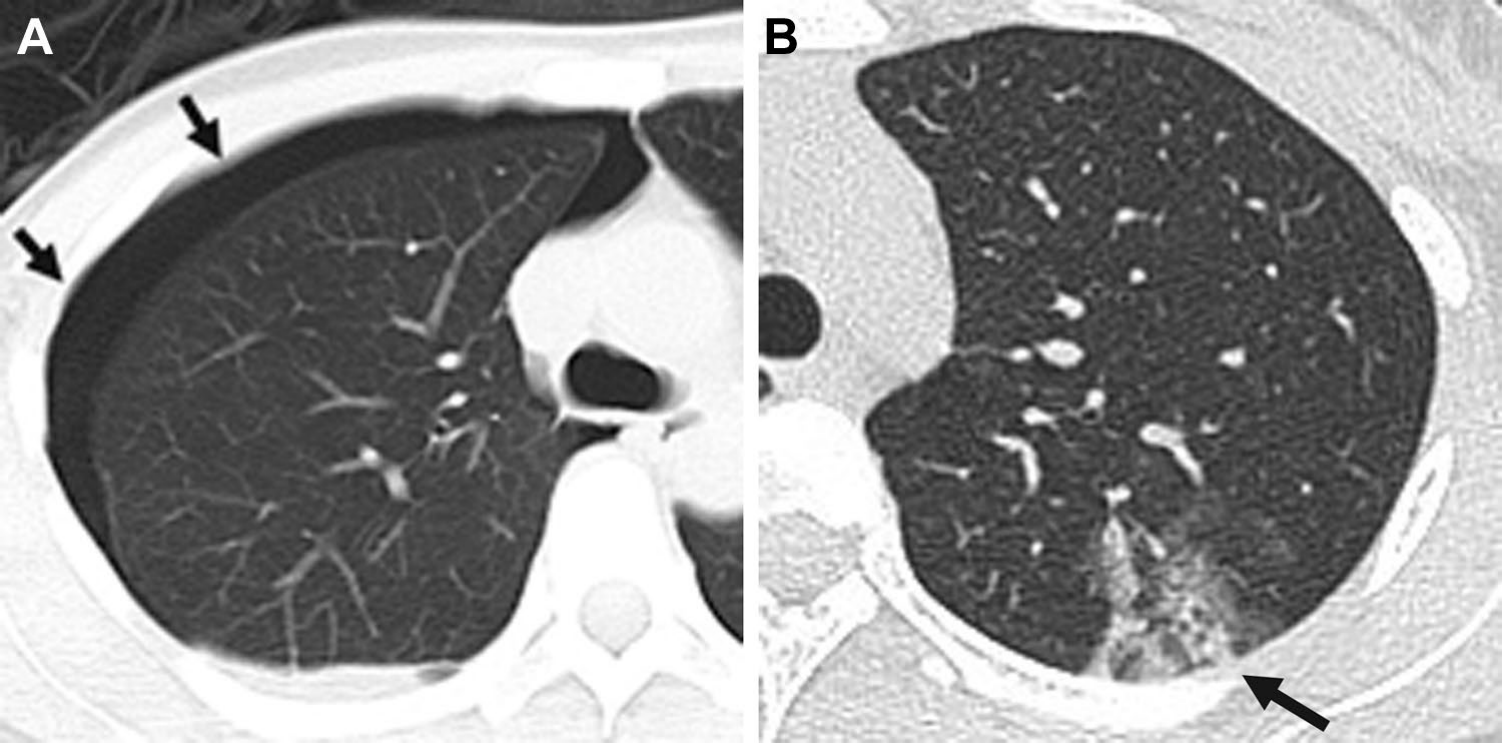
Thoracic endometriosis. A 36-year-old with repeated catamenial right pneumothorax. **A** On CT, right pneumothorax (arrows) is observed. A 28-year-old with repeated catamenial hemoptosis. **B** On CT, patchy ground-glass opacity (arrow) at the left upper lobe reflecting pulmonary hemorrhage is observed

Thoracic endometriosis is rare, and classified as either pleural (Fig. 11A) or parenchymal endometriosis (Fig. 11B) [41, 59–61]. Patients with pleural endometriosis may complain of chest pain and dyspnea due to catamenial pneumothorax (70%) or hemothorax, whereas patients with parenchymal endometriosis usually complain of hemoptysis in the menstrual phase. Pleural endometriosis may appear as T1-high signal intensity nodules at the diaphragm and may be located mostly on the right side and posterior to the vena cava [59]. Pulmonary lesions may appear as patchy ground-glass opacities on CT due to hemorrhage which vary in size during the menstrual cycle and may disappear after the cessation of menstruation.

## Complications

### Ruptured endometrioma

**Fig. 12:**
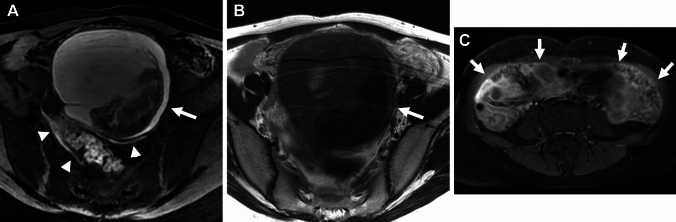
Ruptured endometrioma. A 26-year-old with acute abdomen. MRI is obtained at 3 days after the onset. **A** Fat-saturated T1-weighted image and **B** T2-weighted image show a left endometrioma (arrow) with a lack of tension. High signal intensity peritoneal fluid collection (arrowheads) is revealed on fat-saturated T1-weighted image. Diffuse intense contrast enhancement (arrows) is observed on **C** the post-contrast fat-saturated T1-weighted image reflecting chemical peritonitis

Rupture of endometrioma may occur in 3% of cases, and cause acute chemical peritonitis with severe abdominal pain. Ruptured endometrioma with the absence of tense, flabby, or depressed surface, and hemorrhagic fluid collection in the peritoneal cavity may show T1-high signal intensity clarified on fat-saturated T1-weighted images (Fig. 12A, B) [62, 63]. Strong peritoneal enhancement may be observed on contrast-enhanced images reflecting chemical peritonitis (Fig. 12C).

### Torsion of endometrioma

Torsion of the endometrioma is less common than those of other adnexal masses, possibly due to surrounding adhesions [63]. Patients present acute, intermittent lower abdominal pain. Lack of contrast enhancement of the cyst wall suggests complete torsion of endometrioma.

## Pelvic inflammatory disease

Pelvic inflammatory disease (PID) may be complicated with endometriomas. Infected endometrioma may have thickened walls reflecting fibrous capsules with inflammatory granulation tissue. Usually, tubo-ovarian abscess contains T1-low, T2-high, and DWI-high signal intensity pus, and the admixture of T1-high signal intensity hemorrhagic contents may be suggestive of infected endometrioma [64, 65].

### Peritoneal inclusion cyst

**Fig. 13:**
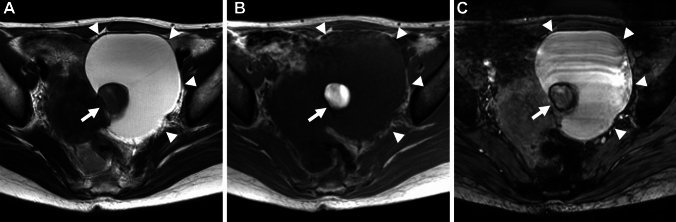
Peritoneal inclusion cyst associated with endometriosis. A 32-year-old with suspicion of a large ovarian cyst on ultrasonography examination. **A** T2-weighted image and, **B** T1-weighted image show a left small endometrioma (arrow) surrounded by a pseudocystic fluid collection (arrowheads) defined by the pelvic wall and pelvic organs. **C** SWAN revealed curved linear signal voids along the wall of the endometrioma

Peritoneal inclusion cysts are localized fluid collection observed in the adhesive pelvis after surgical procedures, trauma, inflammation, or endometriosis. Extensive pelvic adhesions may trap the intraperitoneal fluid and form the pseudocystic lesion. Peritoneal inclusion cyst may appear as a cystic lesion with an irregular margin defined by the adjacent pelvic structures on imaging (Fig. 13) [66].

## Malignant transformation

Endometriomas are associated with a risk of malignant transformation (1% of cases) [67–69]. Endometriosis-associated ovarian carcinoma (EAOC) affects 40–50 years, it is 10–20 year younger than ovarian cancer without endometriosis [67]. Because estrogen may have a role in malignant transformation, patients of reproductive age should be treated, or followed closely. Endometrioma is the precursor lesion for carcinomas via atypical endometriomas through pathways related to oxidative stress, inflammation, and hyperestrogenism and finally to genomic alterations [67, 70]. Persistent oxidative stress induced by endometriosis-dependent hemorrhage may be associated with carcinogenesis [71]. The most frequent histological types of EAOC are endometrioid and clear cell carcinomas [67, 72]. Gene mutation analyses identified oncogenic mutations in endometriosis and normal endometrium and revealed that the same mutations were present in different endometriotic lesions. EAOC may be caused by eutopic endometrial glandular epithelial cells with oncogenic mutations that have undergone menstrual blood reflux and engrafted in the ovary, rather than by low-risk endometriosis acquiring oncogenic mutations and becoming malignant [67, 73].

There are three major pathways for the development of endometriosis-related ovarian neoplasm (ERON). The most major pathway is epithelial malignancies (EAOC) arising by step-wise carcinogenesis, with endometrioid and clear cell carcinomas which are the representative histologic types. Endometrial cells with genetic mutations retrogradely reach the ovary with endometrioma and become cancers promoted by the internal environment of endometrioma. Estrogen receptor-positive cells become endometrioid carcinomas via atypical endometriosis with estrogen stimulation, sometimes occurring bilaterally or with endometrioid carcinoma of the uterine endometrium. On the other hand, estrogen receptor-negative cells become clear cell carcinomas promoted by iron oxidative stress caused by hemorrhagic products in endometriomas and are usually unilateral [70, 74, 75]. The other pathways include other Müllerian-type tumors (seromucinous borderline tumor and mesonephric-like adenocarcinoma) and sarcomas (adenosarcoma, carcinosarcoma, and endometrioid stromal sarcoma). These pathways are rare, and the pathogenesis of endometriosis-associated other Müllerian-type tumors and sarcomas is not well established.

### Imaging criteria of malignant transformation

**Fig. 14:**
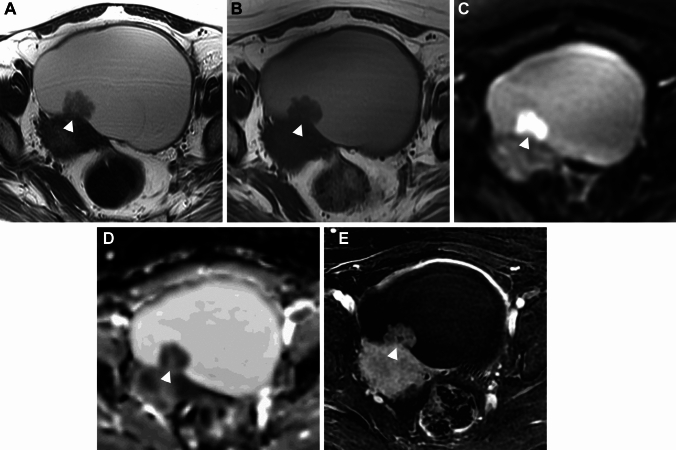
Endometriosis-associated ovarian carcinoma: clear cell carcinoma. A 52-year-old with suspicion of an ovarian cystic mass with mural nodules on ultrasonography examination. **A** T2-weighted image, **B** T1-weighted image, **C** DWI (*b* = 800 s/mm^2^), **D** ADC map, and **E** contrast-enhanced subtraction image show a left ovarian cystic mass with a mural nodule (arrowhead). The cyst contents show high signal intensity on both T1- and T2-weighted images reflecting hemorrhagic fluid. The mural nodule shows intermediate signal intensity on T2-weighted image and water diffusion restriction on DWI, and intense contrast-enhancement on post-contrast images clarified on the subtraction image

**Fig. 15:**
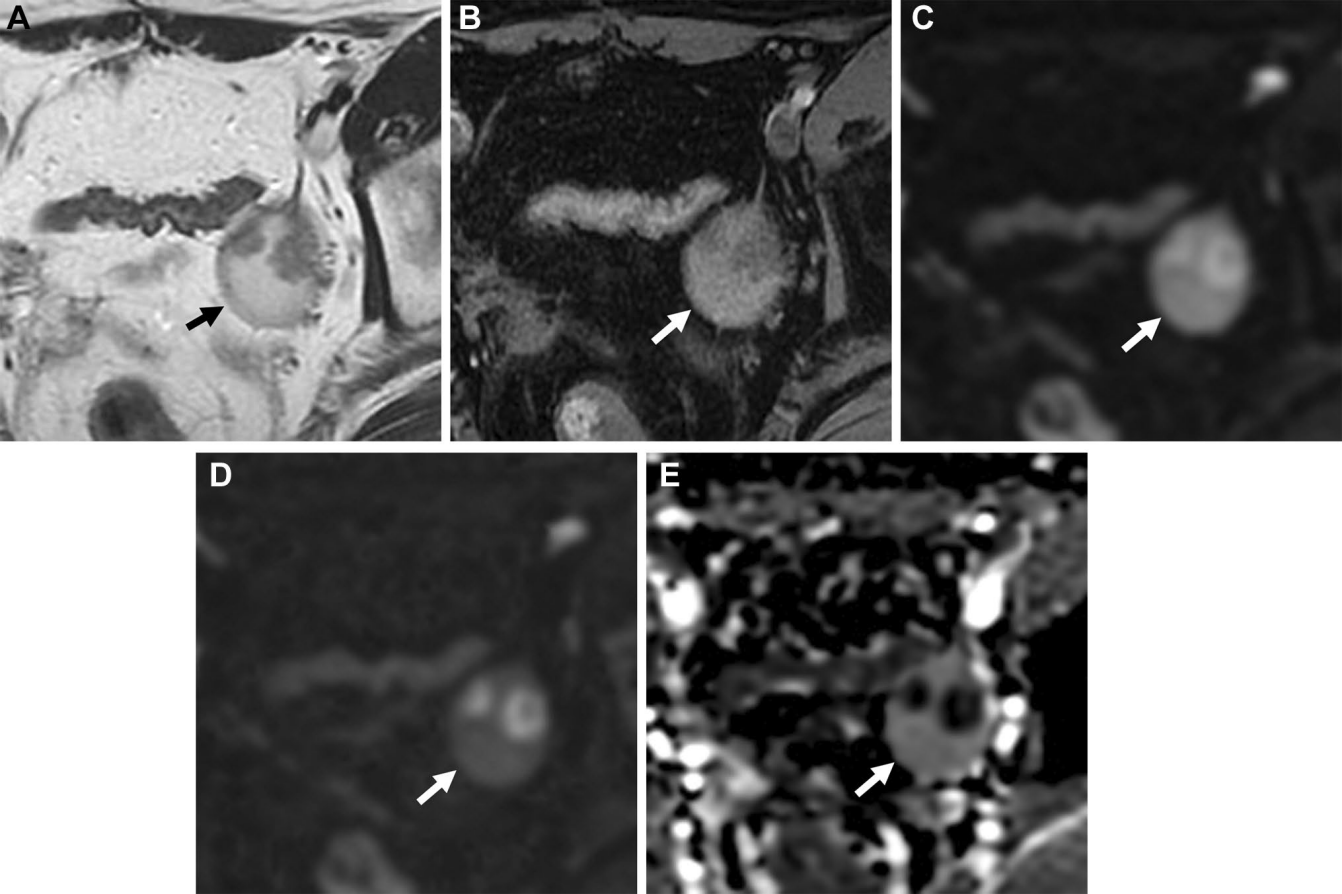
Endometriosis-associated ovarian carcinoma: endometrioid carcinoma. A 35-year-old with suspicion of an ovarian cystic mass with mural nodules on ultrasonography examination. **A** T2-weighted image, **B** fat-saturated T1-weighted image, **C** DWI (*b* = 800 s/mm^2^), **D** computed DWI with high *b* value (*b* = 1500 s/mm^2^), and **E** ADC map show a left ovarian cystic mass (arrow) with mural nodules. The cyst contents show high signal intensity on both T1- and T2-weighted images reflecting hemorrhagic fluid. The mural nodules show water diffusion restriction on DWI, however, high signal intensity hemorrhagic cyst contents mask the signal of mural nodules. High b-value computed DWI can reduce the signal of cyst contents and high signal intensity of mural nodules is clarified

**Fig. 16:**
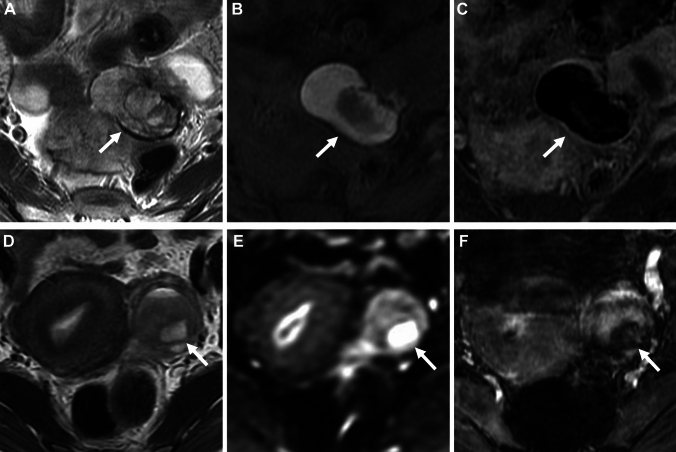
Clots in endometrioma. A 34-year-old with suspicion of an ovarian cystic mass with mural nodules on ultrasonography examination. **A** T2-weighted image, **B** fat-saturated T1-weighted image, and **C** contrast-enhanced subtraction image show a left endometrioma (arrow) with a solid component. The solid component shows intermediate signal intensity on T2-weighted image. The high signal intensity of cyst contents masks the signal of the solid component on the post-contrast image, and no contrast enhancement of the clot is revealed on the contrast-enhanced subtraction image. A 27-year-old with a history of dysmenorrhea. **D** T2-weighted image, and **E** DWI (*b* = 800 s/mm^2^) show a left endometrioma (arrow) with a solid component. The solid component shows intermediate signal intensity on T2-weighted image and water diffusion restriction on DWI, however, appears as a signal void on **F** SWAN suggesting a clot

**Fig. 17:**
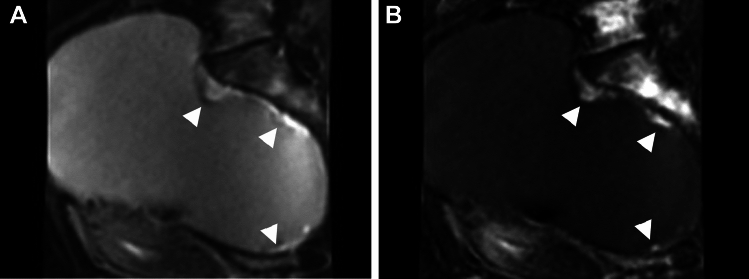
Endometriosis-associated ovarian carcinoma: clear cell carcinoma. A 47-year-old with suspicion of an ovarian cystic mass with mural nodules on ultrasonography examination. **A** On sagittal DWI (reduced field-of-view DWI, *b* = 800 s/mm^2^) a large left endometrioma with small mural nodules (arrowheads) is observed. The high signal intensity of the cyst contents masks the signal of small mural nodules and **B** computed DWI (*b* = 2000s/mm^2^) clarified the high signal intensities of the small mural nodules (arrowheads)

A definite MR finding of malignant transformation is the appearance of contrast-enhanced mural nodules in endometriomas. The contrast-enhancement of the mural nodule may be masked by T1-high signal intensity hemorrhagic fluid, and could be well visualized on contrast-enhancement subtraction images [67, 76, 77]. However, small benign mural nodules may occasionally show contrast enhancement [78]. The malignant mural nodule shows T2-intermediate and DWI-high signal intensities with low apparent diffusion coefficient (ADC) reflecting hypercellularity (Figs. 14, 15), however, clots in the endometrioma may also show DWI-high signal intensity with low ADC mimicking malignant mural nodules (Fig. 16) [79]. The absence of contrast-enhancement of the clots is diagnostic, however, it may be not always clear due to surrounding T1-high signal intensity hemorrhagic fluid. Contrast-enhancement subtraction images are critical for the differentiation by revealing no contrast-enhancement of the clots (Fig. 16C) [77]. SWI may help distinguish the clots, which show low signal intensity reflecting blood products from malignant mural nodules without using contrast materials (Fig. 16F). DWI of hemorrhagic cyst contents in the endometrioma may show high signal intensity, making it difficult to evaluate the signal of mural nodules. High *b* value (≥ 1500 s/mm^2^) computed DWI may be useful for evaluating high signal intensity malignant mural nodules with reduced signal in the cyst contents (Figs. 15, 17).

The disappearance of T2-"shading” and T1-signal decrease due to the dilution by tumor secretion is a suggestive finding of malignant transformation. Other suggestive features of malignant transformation include interval enlargement of the endometrioma and spontaneous reduction in dysmenorrhea due to the reduction of functional endometrial tissue replaced by tumoral tissue [67, 78, 80, 81].

### Seromucinous borderline tumor

**Fig. 18:**
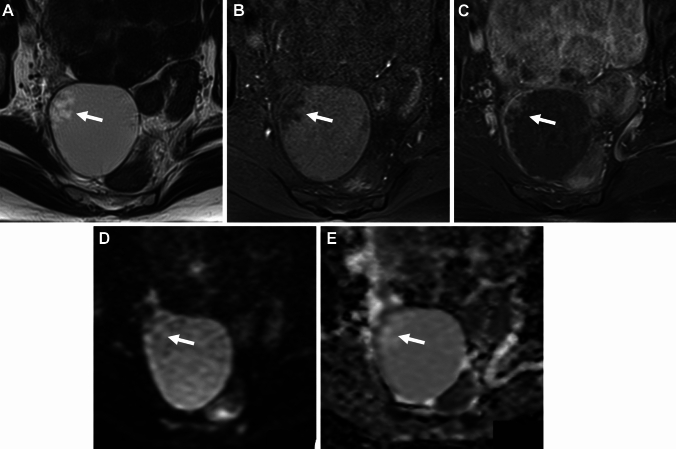
Endometriosis-related ovarian neoplasm: seromucinous borderline tumor. A 55-year-old with suspicion of an ovarian cystic mass with mural nodules on ultrasonography examination. **A** T2-weighted image, **B** fat-saturated T1-weighted image, **C** contrast-enhanced subtraction image, **D** DWI (*b* = 800 s/mm^2^), **E** ADC map show an endometrioma with a papillary mural nodule (arrow). The papillary mural nodule shows high signal intensity with low signal intensity dendritic fibrous core on T2-weighted image, relatively weak contrast-enhancement, and high signal intensity on DWI with high ADC (T2 shine-through)

Seromucinous borderline tumor (SMBT) is an uncommon Müllerian-type tumor arising in endometriomas (at least 1/3 cases). SMBT may affect relatively younger patients (30–40’s) and fertility-preserving surgery may be considered. SMBT may appear as papillary mural nodules within the endometrioma exhibiting T2-high signal intensity, weak contrast-enhancement, and DWI-high signal intensity with high ADC due to T2 shine-through effects reflecting edematous stroma with abundant mucinous material. The mural nodules may contain T2-low signal intensity dendritic fibrous core (Fig. 18) [82–84].

### Mesonephric-like adenocarcinoma

Mesonephric-like adenocarcinoma is a rare Müllerian-type tumor arising in the uterine corpus and ovaries. Histologic and immunohistochemical features overlap with those of cervical mesonephric adenocarcinoma. Mesonephric-like adenocarcinoma may arise from endometriosis as ERON, and affects mostly postmenopausal patients with aggressive behavior [85, 86].

### Sarcomas

Carcinosarcoma is a rare ovarian tumor (2% of ovarian malignancies), which is a biphasic neoplasm composed of high-grade malignant epithelial and mesenchymal elements. Carcinosarcoma may arise from endometriosis, mostly with endometrioid carcinoma, and exhibit as a large mass with stained-glass appearance, hemorrhage, and necrosis on MRI [87]. Adenosarcoma is a rare neoplasm of low-grade malignancy that consists of an admixture of sarcomatous mesenchymal and benign glandular epithelial components. It arises most commonly in the uterine endometrium, but may also occur in the ovaries, and extragenital sites in association with endometriosis [88]. Endometrial stromal sarcoma is a rare malignant uterine tumor originating from endometrial stromal cells. Endometrioid stromal sarcoma may originate primarily from extra-uterine sites such as the ovaries, peritoneal cavity, retroperitoneum, and vagina associated with endometriosis [89].

### *Clear cell carcinoma *via* adenofibroma*

Ovarian cancer may arise from benign adenomas or adenofibromas as step-wise carcinogenesis. Especially, a solid variant of clear cell carcinoma (CCC) may arise from non-cystic endometriosis via clear cell adenofibroma (CCAF) as the CCAF–CCC sequence [90, 91]. Clear cell adenofibroma components co-exist in 15–21% of clear cell carcinomas and show lower tumor grade and better prognosis. The malignant focus of clear cell carcinoma arising from clear cell adenofibroma shows DWI-high signal intensity with low ADC and intense early and prolonged contrast enhancement [92].

## Endometriosis-associated tumor-like lesions

### Decidualized endometrioma during pregnancy

**Fig. 19:**
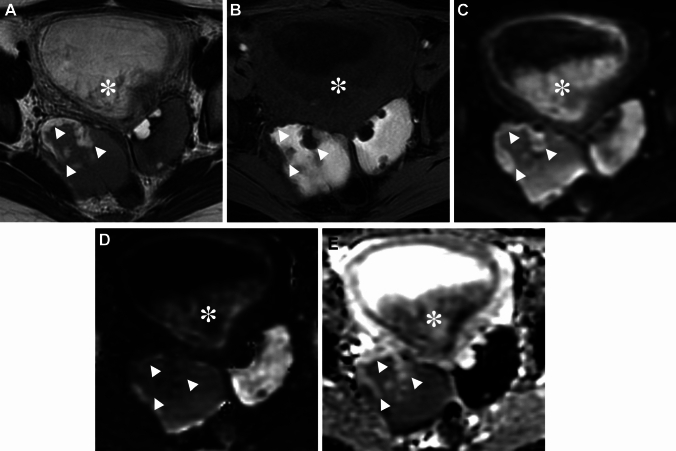
Decidualized endometrioma. A 32-year-old pregnant (13 weeks) with suspicion of an ovarian cystic mass with mural nodules on ultrasonography examination. **A** T2-weighted image, **B** fat-saturated T1-weight image, **C** DWI (*b* = 800 s/mm^2^). **D** computed DWI (*b* = 2000s/mm^2^), and **E** ADC map show a right endometrioma with multiple, flat mural nodules (arrowheads) exhibiting T2-prominent high signal intensity, T1-low signal intensity, and high signal intensity on DWI with high ADC (T2 shine-through) similar to those of the placenta (*). On the high b-value (*b* = 2000s/mm^2^) computed DWI shows the signal decrease of the mural nodules

With the hypertrophy of the endometrial stromal cells, the normal uterine endometrium may thicken and transform into the decidua induced by progesterone during pregnancy. This phenomenon may also occur in ectopic endometrial tissue such as endometrioma. Decidualized endometrioma may manifest as broad-based, flat or polypoid mural nodules with smooth contours. Signal intensity is similar to that of the placenta: T2-prominent high signal intensity and DWI-high signal intensity with high ADC (T2 shine-through) reflecting edematous, vascularized decidualized tissue. The ADC measurement is useful for differentiating decidualized nodules from malignant transformation (Fig. 19) [93, 94]. Computed DWI with high *b* values (*b* ≥ 1500 s/mm^2^) can distinguish decidualized endometriomas from ovarian cancers by visual evaluation. Decidualized mural nodules show signal decrease on computed DWI with high *b* values (Fig. 19D), whereas high signal of malignant mural nodules is maintained [95].

Because both decidualized endometrioma and SMBT may appear as T2-high signal intensity mural nodules in the endometriomas, differentiation becomes problematic if the tumor is detected during pregnancy. Both lesions show DWI-high signal intensity with high ADC (T2 shine-through), and morphological appearances may be the clues for the differential diagnosis. The greater number and lower height are suggestive of decidualized endometrioma, whereas the lobulated margin, pedunculated configuration, and T2-low signal intensity core (the reported frequency ranges from 43 to 61%) of mural nodules are suggestive of SMBT [84, 96].

### Polypoid endometriosis

**Fig. 20:**
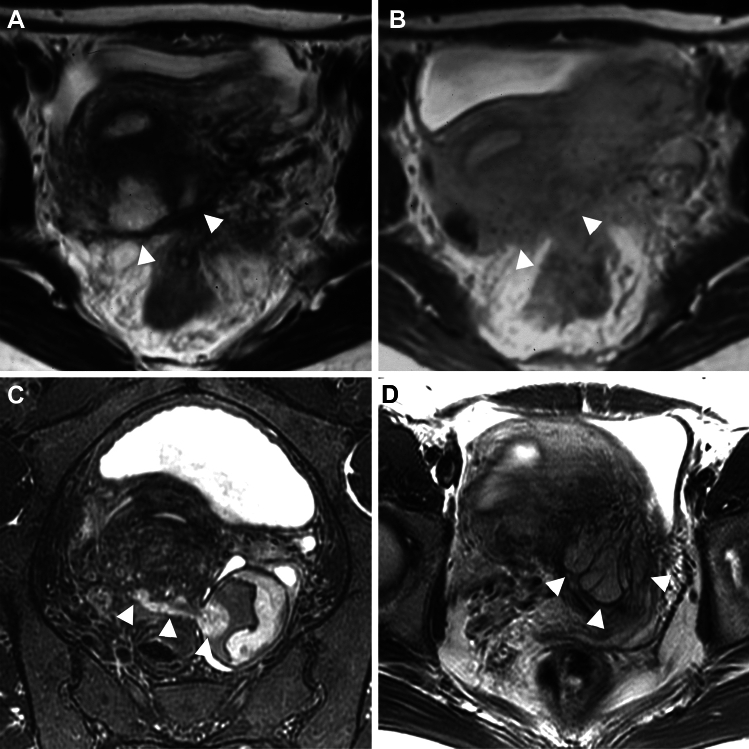
Polypoid endometriosis. A 47-year-old with lower abdominal pain associated with genital bleeding and suspicion of deep endometriosis. **A** T2-weighted image and **B** post-contrast T1-weighted image show polypoid masses (arrowheads) exhibiting T2-high signal intensity and intense contrast-enhancement protruding to the posterior wall of the uterine body with adenomyosis. The masses are surrounded by T2-low signal intensity adhesive fibrous tissue as “black rim sign”. The fibrous rim also shows intense contrast enhancement. A 33-year-old with a history of hypermenorrhea and dysmenorrhea and suspicion of deep endometriosis. **C.** Oblique coronal fat-saturated T2-weighted image shows a left endometrioma with high signal intensity mural nodule that extends to the Douglas' pouch (arrowheads). A 30-year-old with a history of dysmenorrhea and suspicion of deep endometriosis. **D** T2-weighted image shows polypoid masses infiltrating into the myometrium (arrowheads)

Polypoid endometriosis is a rare variant of endometriosis with histological features resembling those of endometrial polyps. Polypoid endometriosis frequently affects perimenopausal women and hormonal factors such as unopposed estrogen therapy or tamoxifen use may play a role in its pathogenesis and forms large, often multiple polypoid masses simulating malignancy [97]. Polypoid endometriosis may arise within the endometrioma mimicking malignant transformation, or exist in the pelvic cavity protruding to adjacent structures simulating peritoneal carcinomatosis. The edematous endometriotic tissue may show T2-high signal intensity and DWI-high signal intensity with high ADC (T2 shine-through), and intense contrast enhancement like an endometrial polyp (Fig. 20) [98]. Characteristic surrounding T2-low signal intensity adhesive fibrous tissue in the peritoneal lesions as “black rim” sign suggests its deep endometriosis origin (Fig. 20A) [98, 99]. Polypoid endometriosis may often show areas of hyperplasia and rarely cause malignant transformation, which is usually, endometrioid carcinoma. A reported case of peritoneal polypoid endometriosis with malignant transformation showed T2-high signal intensity and weak contrast enhancement similar to the signal pattern of well-differentiated endometrial carcinoma of the uterus [100].

## Conclusions

In evaluating the severity and location of the endometriosis (endometrioma, deep endometriosis, and extra-genital endometriosis), and for the diagnosis of various complications of endometriosis, malignant transformation, and endometriosis-related tumor-like lesions MRI including advanced MR techniques is a non-invasive tool that is feasible for the diagnostic strategy by its good tissue contrast and multiplanar capability.
